# Cooperativity of Negative Autoregulation Confers Increased Mutational Robustness

**DOI:** 10.1103/PhysRevLett.116.258104

**Published:** 2016-06-22

**Authors:** David C. Marciano, Rhonald C. Lua, Christophe Herman, Olivier Lichtarge

**Affiliations:** 1 Department of Molecular and Human Genetics, Baylor College of Medicine, Houston, Texas 77030, USA; 2 Verna and Marrs McLean Department of Biochemistry and Molecular Biology, Baylor College of Medicine, Houston, Texas 77030, USA; 3 Computational and Integrative Biomedical Research Center, Baylor College of Medicine, Houston, Texas 77030, USA

## Abstract

Negative autoregulation is universally found across organisms. In the bacterium *Escherichia coli*, transcription factors often repress their own expression to form a negative feedback network motif that enables robustness to changes in biochemical parameters. Here we present a simple phenomenological model of a negative feedback transcription factor repressing both itself and another target gene. The strength of the negative feedback is characterized by three parameters: the cooperativity in self-repression, the maximal expression rate of the transcription factor, and the apparent dissociation constant of the transcription factor binding to its own promoter. Analysis of the model shows that the target gene levels are robust to mutations in the transcription factor, and that the robustness improves as the degree of cooperativity in self-repression increases. The prediction is tested in the LexA transcriptional network of *E. coli* by altering cooperativity in self-repression and promoter strength. Indeed, we find robustness is correlated with the former. Considering the proposed importance of gene regulation in speciation, parameters governing a transcription factor’s robustness to mutation may have significant influence on a cell or organism’s capacity to evolve.

Most biological systems are known to be robust to environmental variations, cell-to-cell variations and mutational perturbations [1,2]. Robustness contributes to the survival of the organism, and may also allow for the accumulation of phenotypically silent genetic variation [3–7], which, when exposed by a major perturbation or stress, can be a substrate for selection [1].

A network-level mechanism that can contribute to robustness of an organism is a negative feedback loop or autoregulation. Negative autoregulation (NAR) in gene regulatory networks is universally found across organisms. In *E. coli*, the NAR network motif is exhibited in about 40% of known transcription factors (TF) [8]. NAR may have arisen in evolution to speed the response time of gene circuits [9–11], to increase the stability of gene expression against low-frequency noise [12,13], and to linearize or increase the input dynamic range of the gene’s input-output response curve [8,14]. The latter two functions of NAR can be viewed as providing gene expression stability against transient perturbations in biochemical parameters.

Only recently have experiments been devised showing that NAR buffers against the effect of mutations. In yeast, NAR has been shown to stabilize expression levels of theROX1TF as a result of natural genetic variations across strains [15], suggesting that NAR does confer robustness to mutations. In the mammalian visual system, genetic perturbations that significantly alter the deactivation of photoexcited rhodopsin in rod photoreceptor cells do not much alter the amplitude of single-photon responses [16]. However, the same perturbations, when crossed into a line wherein calcium-mediated negative feedback is absent, produced larger alterations in single-photon responses, showing that negative feedback enables a more robust, stereotyped response that promotes reliable signal transmission through the visual pathway. In more recent work in bacteria, NAR was shown to impart robustness to TF mutations and stabilize expression levels [17]. However, it is an open question as to which components of NAR can influence mutational robustness.

Here we examine a model of a gene circuit with a TF, under NAR, that regulates the expression of a target gene (Fig. 1). In this model, negative regulation of protein production by a TF is specified by a Hill function with parameters that can be altered by mutation. We show that the steady-state level of the target gene product is robust or stable against variations in either the TF degradation rate (*β_r_*) or strength of binding between the TF and the promoters (*K_r_* and *K_g_*) when the TF is under NAR, compared with no feedback. We also predict and experimentally confirm that this robustness improves as the degree of cooperativity (Hill coefficient, *h_r_*) of the TF regulation of its own expression increases. The results suggest that a selective pressure for robustness may drive the evolution of negative feedback loops in transcription networks.

## Conditions satisfied in the steady-state by TF and target gene levels

The steady-state levels of the TF (*r*) and the target gene (*g*) are reached when protein production is balanced by protein degradation (rates *β_r_* and *β_g_*). With negative feedback present,

(1) $$\beta_{r}r=\frac{\alpha_{r}}{1+{\left(r/K_{r}\right)}^{h_{r}}}.$$

On the right-hand side of this equation, the repression of protein production by the TF is modeled as a Hill function [35]. Without feedback (setting *h_r_* = 0), we have *β_r_r* = *α_r_*/2. The Hill coefficient *h_r_* suggests the degree of cooperativity in the regulation of gene expression. The Hill repression coefficient *K_r_* is the TF concentration at which the protein production rate is reduced to half the maximum (rate *α_r_*).

The dependence of the target gene concentration on the repressor concentration is the same with or without feedback

(2) $$\beta_{g}g=\alpha_{g}G(r/K_{g}).$$

The function *G*(*x*) may take the form of a Hill function [35,36]

(3) $$G(r/K_{g})={\left[1+{\left(r/K_{g}\right)}^{h_{g}}\right]}^{-1},$$

where *h_g_* > 0 corresponds to repression, and *h_g_* < 0 corresponds to activation.

## Target gene expression becomes less sensitive to changes in degradation rate β̃_r_ and repressor binding K̂_r_ as the Hill coefficient h_r_ increases

In order to examine the effect of varying the degradation rate *β_r_* while all other parameters are held fixed, we use dimensionless variables and put *r̃* = *r*/*K_r_*, *β̃_r_* = [*β_r_*/(*α_r_*/*K_r_*)], *g*′ = [*g*/(*α_g_*/*β_g_*)]. With negative feedback present,

(4) $$\tilde{r}=\frac{1/{\tilde{\beta }}_{r}}{1+{\tilde{r}}^{h_{r}}}.$$

Without feedback, we have *r̃* = 1/2*β̃_r_*. The dependence of the target gene concentration on the repressor is the same with or without feedback:

(5) $$g^{\prime }=G\left(\frac{\tilde{r}}{K_{g}/K_{r}}\right).$$

In order to examine the effect of varying the repression coefficient *K_r_*, we put *r̂* = [*r*/(*α_r_*/*β_r_*)], *K̂_r_* = [*K_r_*/(*α_r_*/*β_r_*)], *K̂_g_* = [*K_g_*/(*α_r_*/*β_r_*)], *g*′ = [*g*/(*α_g_*/*β_g_*)]. With negative feedback present,

(6) $$\hat{r}={\left[1+{\left(\hat{r}/{\hat{K}}_{r}\right)}^{h_{r}}\right]}^{-1}.$$

Without feedback, we have *r̂* = 1/2. The dependence of the target gene concentration on the repressor is the same with or without feedback:

(7) $$g^{\prime }=G(\hat{r}/{\hat{K}}_{g}).$$

To show that the functional dependence of *g*′ on *K̂_r_* is the same as that of *g*′ with *β̃_r_*, multiply both sides of Eq. (6) by 1/*K̂_r_*,

(8) $$\hat{r}/{\hat{K}}_{r}=\frac{1/{\hat{K}}_{r}}{1+{\left(\hat{r}/{\hat{K}}_{r}\right)}^{h_{r}}}.$$

Using *r̂*/*K̂_r_* = *r̃*, the preceding equations become

(9) $$\tilde{r}=\frac{1/{\hat{K}}_{r}}{1+{\tilde{r}}^{h_{r}}},\;\;\;g^{\prime }=G\left(\frac{{\hat{K}}_{r}\;\tilde{r}}{{\hat{K}}_{g}}\right)=G\left(\frac{\tilde{r}}{K_{g}/K_{r}}\right),$$

which are identical to Eqs. (4) and (5) if *K̂_r_* is identified as *β̃_r_* and *K_g_*/*K_r_* is constant.

**FIG. 2:**
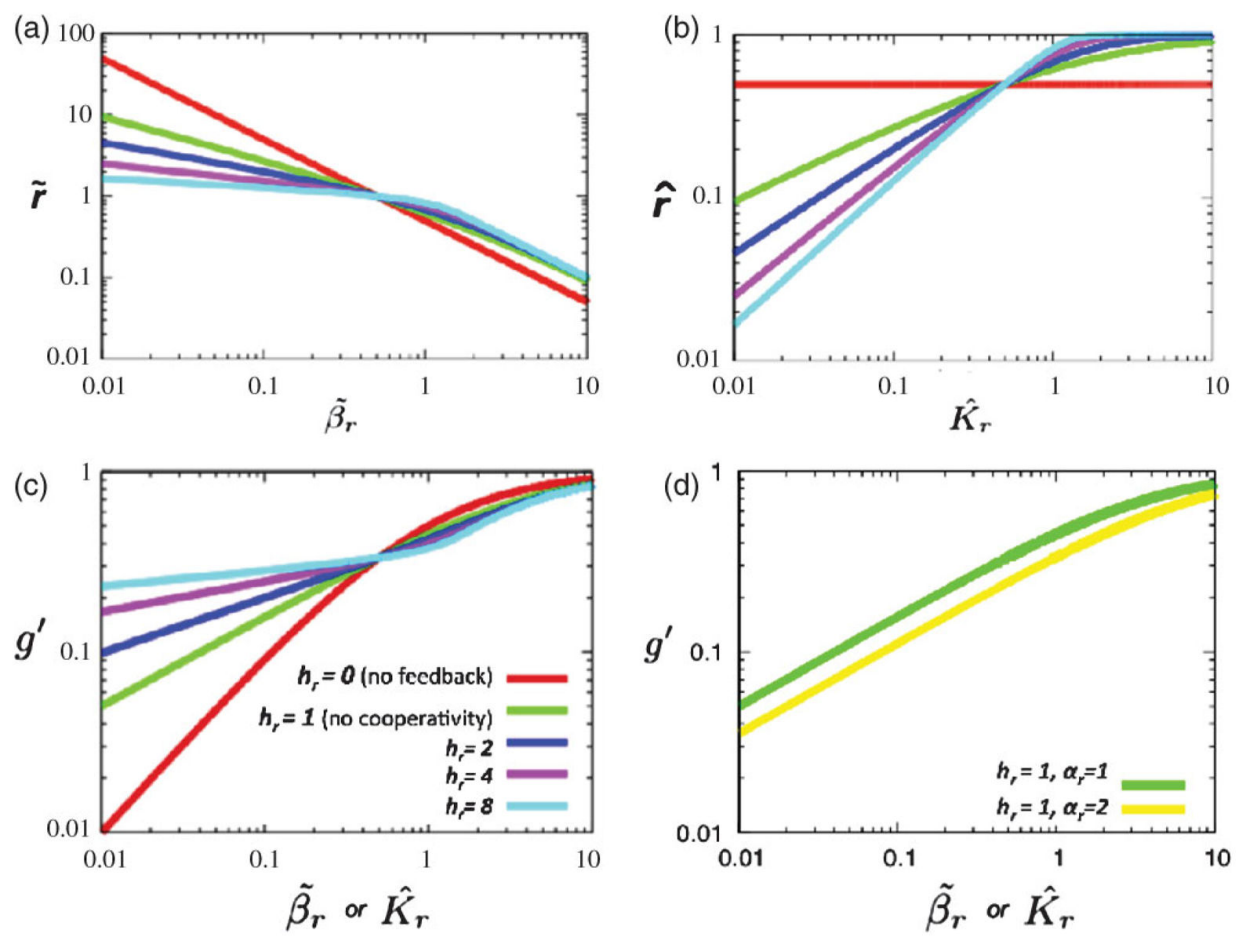
Variation of *g* and *r* with *β_r_*, *K_r_*, and *h_r_*. (a) TF concentration *r̃* against TF degradation rate (*β̃_r_*) for different values of the Hill coefficient *h_r_*. (b) TF concentration *r̂* against the Hill repression coefficient (*K̂_r_*) for different values of the Hill coefficient *h_r_*. (c) Normalized target gene concentration *g*′ against *β̃_r_* or *K̂_r_* for different values of the Hill coefficient *h_r_*, showing that *g*′ becomes more robust to TF mutations manifested as variations in either *β̃_r_* or *K̂_r_* as *h_r_* increases. *g*′ is calculated using Eq. (3) with *h_g_* = 1 (repression), *K_g_* = 0.5*K_r_*. The slope at the intersection of the curves is 2[3(1 + *h_r_*/2)]^−1^ (see Eq. S.8, [18]). (d) *g* decreases when *α_r_* increases. The new values lie on a new curve that is shifted to the right.

Figure 2 plots the target gene concentration *g*′ against *β̃_r_* at several values of *h_r_*, where *g*′ is calculated numerically from Eqs. (4), (5), and (3). The exact same set of curves are obtained when *g*′ is plotted against *K̂_r_*. The calculation of the parameter sensitivity coefficients, which give the slope of these curves and which quantify robustness, are presented in the Supplemental Material [18]. Figure 2(d) also demonstrates the effect of increasing *α_r_* on *g*′. The new values lie on a curve shifted to the right, which lowers *g*′, but does not decrease the parameter sensitivity coefficient and the coefficient of variation. These results show that target gene expression becomes less sensitive to perturbations in either degradation rate of the TF or its affinity to the promoter as the strength of negative feedback increases through *h_r_*.

## Experimental validation

**FIG. 3:**
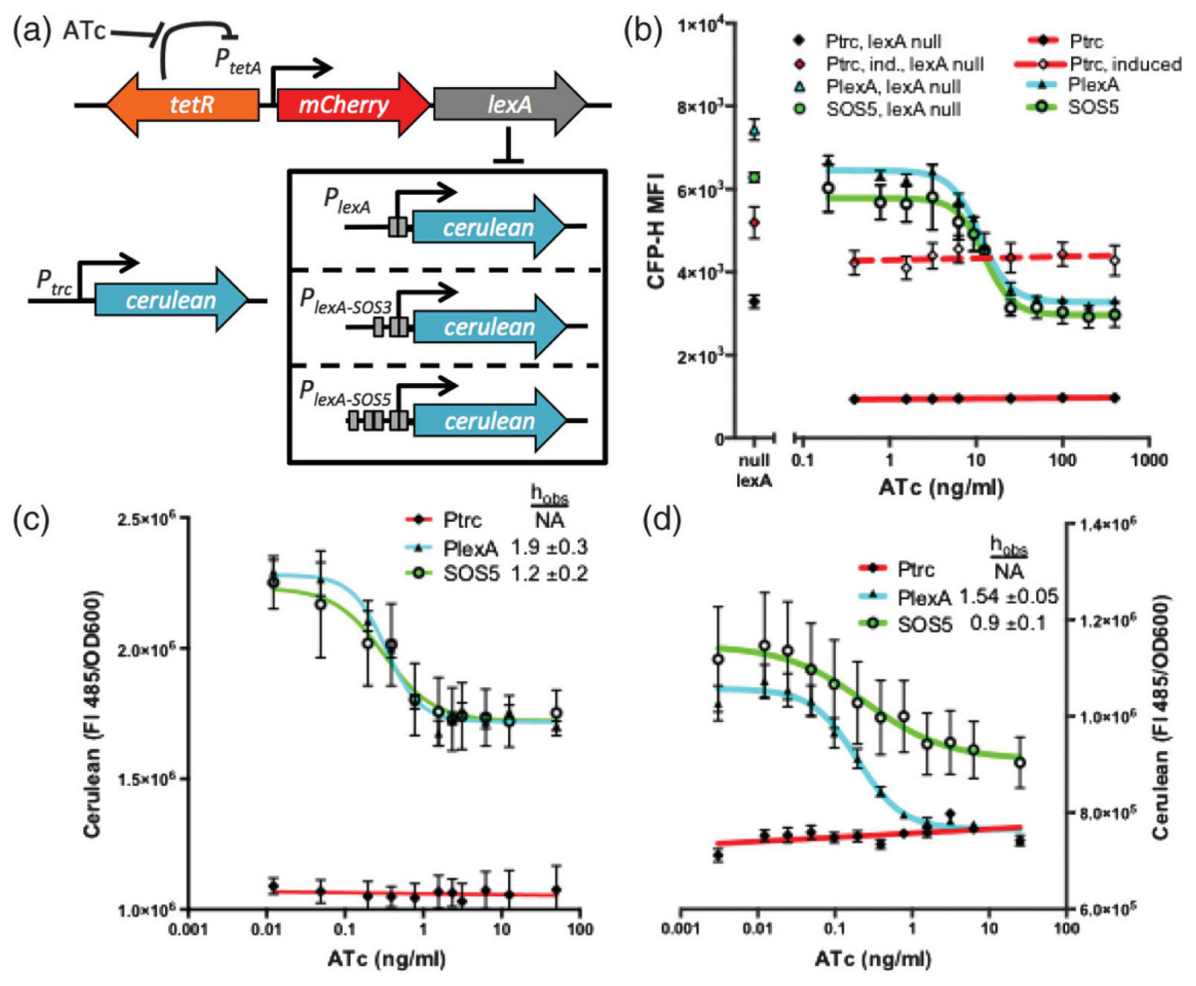
Experimental modification and characterization of the Hill function. (a) Schematic of the genetic system with TetR control of *lexA*. (b) ATc dose-dependent transcription from *P_trc_*, *P_lexA_*, and *P_lexA_*_–SOS5_ as inferred from cerulean fluorescence during flow cytometry. Symbols next to the *y* axis indicate promoter activity in a null-*lexA* strain. *P_lexA_* and *P_lexA_*_–SOS5_ were grown in rich media and 30ng/mL MMC (mitomycin C, which induces the cleavage of LexA); median fluorescence intensity (MFI). (c),(d) ATc dose-dependent cerulean fluorescence measured in a plate reader in the absence (d) or presence of 120ng/mL MMC (c). Panels (b)–(d) taken together show that the Hill coefficient in *P_lexA_* is greater than in *P_lexA_*_–SOS5_.

We used the *E. coli* LexA repressor of the SOS DNA damage response as a model system because it displays negative feedback by repression of its own promoter [37]. However, in order to characterize the *lexA* promoter (*P_lexA_* and variants), we needed to break the negative feedback loop to gain control over LexA expression, determine *P_lexA_* activity over a range of LexA protein levels and fit the Hill equation. Replacing the *lexA* promoter on the chromosome with an anhydrotetracycline (ATc)-inducible *tetA* promoter (*P_tetA_*) facilitates control over LexA levels [Fig. 3(a)]. Dose-dependent transcription of *lexA* is inferred by measuring the fluorescence of the co-transcribed red fluorescent protein variant, mCherry (Fig. S.3, [18]). A plasmid-encoded *P_lexA_* is reintroduced as a fusion to a cyan fluorescent protein variant, *cerulean* [Fig. 3(a)]. *P_lexA_* has two SOS boxes and, although not experimentally quantified, it has been reported to have moderate cooperativity [17,36,38]. By adding more SOS boxes (*P_lexA_*_–SOS3_ and *P_lexA_*_–SOS5_), we expected to increase the cooperativity. A completely different promoter with 0 SOS boxes (*P_trc_*) is a control system that is not repressed by LexA and should be unresponsive to changes in LexA levels. By deconstructing the *lexA* gene circuit into a simpler repression system we can experimentally measure parameters of *P_lexA_* and variants thereof.

To estimate *α_r_*, we created a strain where LexA is deactivated (null-*lexA*) and measured the *lexA* promoter activity through the fluorescence of *cerulean*. Unexpectedly, *α_r_* varied substantially between the promoter variants. It was largest for *P_lexA_* [MFI (7.44 ± 0.25) × 10^3^ a.u.], less for *P_lexA_*_–SOS5_ [MFI (6.28 ± 0.12) × 10^3^] and *P_trc_*-induced [MFI (5.19 ± 0.38) × 10^3^], and least for *P_trc_* [MFI (3.29 ± 0.16) × 10^3^]. Multiple experiments suggested that *P_lexA_*_–SOS3_ [MFI (1.02 ± 0.06) × 10^4^; Fig. S.4, [18]] is too unstable for us to make meaningful conclusions from this variant. These differences in *α_r_* may influence mutational robustness.

By fitting a Hill function to the plots of fluorescence data against ATc, we can estimate the cooperativity of each promoter construct (Figs. S.3 and S.4 in the Supplemental Material [18]). The calculated Hill coefficient observed (*h*_obs_) for *P_lexA_* of 1.5 ± 0.2 in this system agrees with previous estimates of LexA cooperativity ranging from 1 to 2.4 [17,36,38]. Surprisingly, addition of three SOS boxes upstream of the RNA polymerase binding site in the *P_lexA_*_–SOS5_ construct had the effect of lowering *h*_obs_ to 1.2 ± 0.2. However, the ATc-inducible system may fall short of characterizing the Hill function at low LexA levels. In theory, the cerulean fluorescence levels (CFP-H) obtained in the null-*lexA* strain should be nearly equivalent to fluorescence levels observed at low ATc concentrations wherein LexA expression is fully repressed by TetR. However, cerulean levels in the null-*lexA* strain are actually 2 to 4 times higher than observed when TetR is fully repressing LexA expression in the absence of ATc. Therefore, we sought to further reduce LexA levels in the ATc-inducible system by activating a LexA coprotease, RecA, with mitomycin C [39]. By plotting fluorescence data against ATc under different growth conditions [Figs. 3(b)–(d)], we sample different ranges of LexA levels with Fig. 3(b) being at the lower end and Fig. 3(d) being at the higher end. We interpret the crossing between the Hill curves as a difference in the Hill coefficient. Overall, we find both *α_r_* and *h*_obs_ to be reduced in *P_lexA_*_–SOS5_ relative to *P_lexA_* in multiple assay conditions. This provides us the means to test the hypothesis that feedback strength, as determined by *α_r_* and *h*_obs_, influences mutational robustness.

**FIG. 4:**
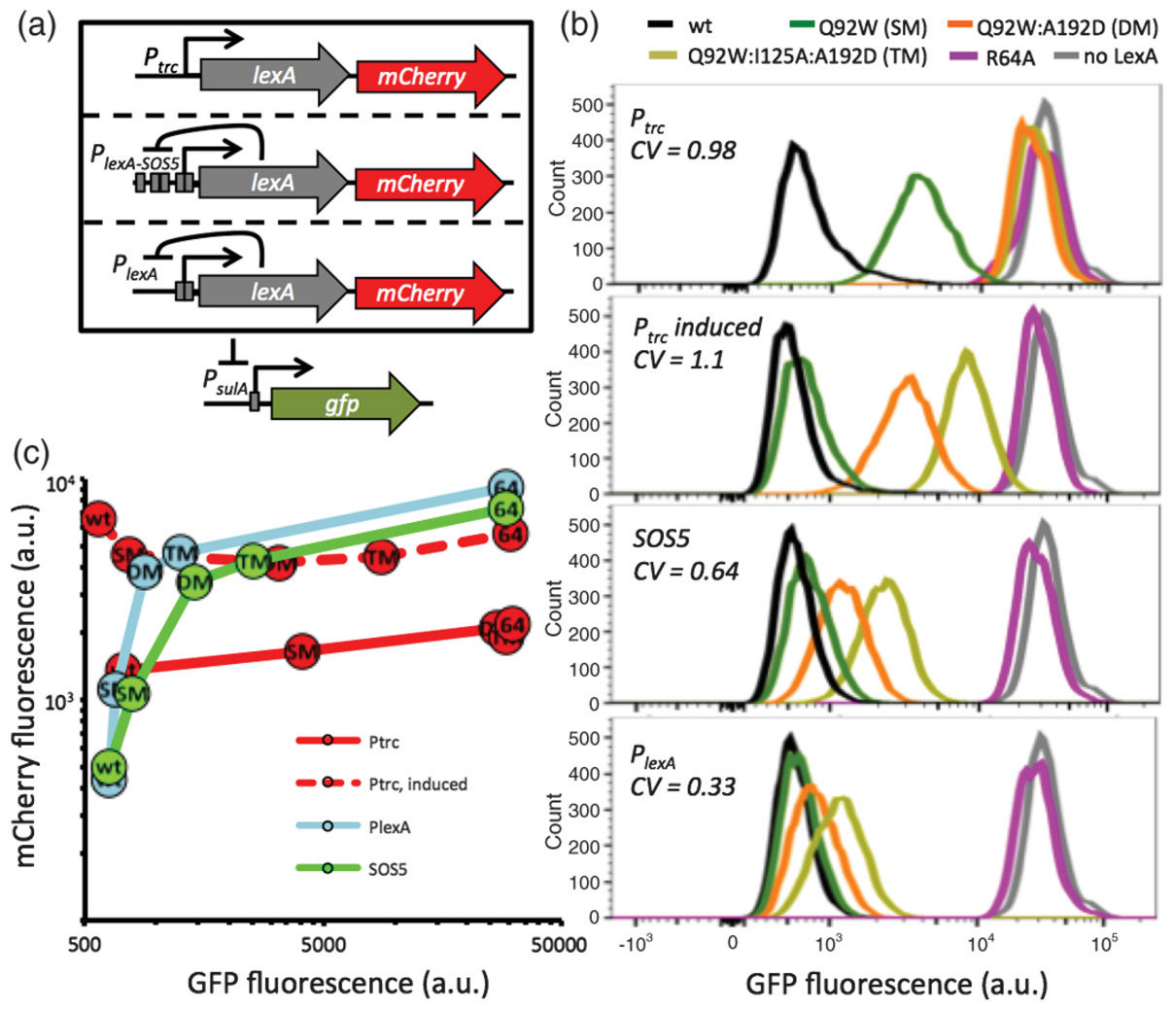
Influence of feedback strength on LexA mutational robustness. (a) Schematic of the genetic system. (b) Representative flow cytometric data of GFP fluorescence distributions for each promoter construct. Coefficient of variation (*CV*) using wt, SM, DM, and TM is taken as a measure of mutational robustness. (c) mCherry MFI is plotted against GFP MFI for each promoter construct. Each data point is the average MFI of either wt, Q92W (SM), Q92W:A192D (DM), Q92W:I125A:A192D (TM) or R64A (64) LexA taken across four biological replicates. Error bars representing the standard error of the mean are smaller than the data point labels.

In order to determine how the Hill function influences mutational robustness of the LexA gene circuit, we generated a panel of plasmids that place *lexA-mCherry* under control of either “no feedback” (*P_trc_*), “NAR weak” (*P_lexA_*_–SOS5_) or “NAR strong” (*P_lexA_*) [Fig. 4(a)]. The presence of mCherry downstream of *lexA* permits inference of promoter activity through measurement of mCherry fluorescence levels. This plasmid panel was transformed into *E. coli* cells with a chromosomally encoded *P_sulA_*-*gfp* reporter that is repressed by LexA to allow assessment of LexA repressor function via monitoring of GFP fluorescence levels. Next, we progressively introduced specific mutations into the *lexA* gene that severely attenuate repressor function in the cell by increasing degradation rate [17,40]. Any one of these substitutions is highly deleterious and their combination would be expected to render the LexA function irrecoverable. We also generated a R64A single mutant in the DNA binding domain that renders LexA unable to repress gene expression in order to measure the maximal expression from the *P_lexA_*, *P_lexA_*_–SOS5_, and *P_sulA_* promoters.

NAR rescues the repressor function from deleterious mutations and feedback strength further enhances this rescue [Fig. 4(b) and Table S.2 [18]]. As expected, in the absence of NAR, the Q92W mutant greatly attenuates LexA’s ability to repress *P_sulA_*-*gfp*, and introducing additional deleterious mutations increases *P_sulA_*-*gfp* expression to the point of being nearly indistinguishable from the empty vector control system that is devoid of LexA [Fig. 4(b), *P_trc_*]. Although simply raising LexA expression levels in the absence of NAR can rescue the Q92Wmutant and lower GFP levels in others, the coefficient of variation (*CV*) taken across wt, SM, DM, and TM mutants is not diminished [Figs. 4(b) *P_trc_*-induced and 2(d)]. This indicates that increasing *α_r_* alone is not sufficient to significantly improve overall mutational robustness of this system. In the context of the “weak” negative feedback promoter (*P_lexA_*_–SOS5_), these LexA mutants partially regain the cellular capacity to repress *P_sulA_*-*gfp* [Fig. 4(b) SOS5]. With stronger negative feedback, these mutants are further rescued [Fig. 4(b), *P_lexA_*] and the *CV* is even lower. Simultaneously monitoring mCherry fluorescence levels allows us to determine how each promoter responds to the introduction of the LexA mutations. As expected, mCherry levels are flat across the LexA mutants in the *P_trc_* construct lacking feedback [Fig. 4(c)]. In contrast, the feedback constructs are able to increase LexA expression in step with progressively deleterious mutations. These experimental data indicate a role for cooperativity in providing additional mutational robustness in target gene repression by increasing expression levels of the mutated transcriptional repressor.

## Negative feedback TFs are more frequently mutated than other groups of genes

In order to find further evidence for the influence of negative feedback on mutational robustness, we turned to the long-term experimental evolution project of the Richard Lenski lab. At 40 000 generations of serial passaging [41], we found 16 out of 68 negative feedback TF genes [42] have nonsynonymous mutations while just 10 out of 93 TFs without negative feedback have these mutations (*χ*^2^ test *p* = 0.030). More broadly, across all 4290 genes, 621 of which are mutated, the 68 negative feedback TFs are more frequently mutated than expected (*χ*^2^ test *p* = 0.032). This suggests a role for negative feedback in shaping the mutational landscape of an evolving bacterial population.

## Conclusion

Negative feedback imparts robustness to changes in most system parameters, but not all [43]. Changing the strength of a repressor’s self-repression (*K_r_*) results in a significant change of repressor protein level and higher cooperativity increases the sensitivity of repressor protein levels (*r*) to changes in *K_r_* [Fig. 2(b)]. And yet, downstream gene repression still remains insulated against changes in *K_r_* so long as the repressor’s affinity for the target promoter (*K_g_*) changes in step [Fig. 2(c)]. Changing both *K_r_* and *K_g_* simultaneously may be achieved by amino acid mutations in the repressor that directly affect DNA affinity. Mutations reducing DNA affinity result in elevated protein levels of the mutant repressor and repression of target promoters can be maintained because repressor concentration in the cell has shifted to match the change in *K_g_*. However, mutations that specifically change *K_r_*, such as a substitution in the DNA recognition site of the repressor’s promoter, will elevate expression of a fully functional repressor and have a disproportionate repression effect on other genes whose affinities for the repressor are unchanged. Therefore, which parameter(s) a mutation alters determines how the negative feedback network responds.

Previous theoretical and computational work noted the influence of cooperativity on NAR’s suppression of variability. Using a power-law formalism and sensitivity analysis, it was shown that robustness to changes in the degradation rate is enhanced by NAR of regulator gene expression [44] and this robustness increases with kinetic order. This kinetic order corresponds to a Hill coefficient in the limit of strong regulator-promoter binding. Other work combining stochastic modeling with parameter variation likewise noted the effect of NAR cooperativity in buffering repressor levels against changes in degradation rate [45,46]. We build upon this work by showing that changes in *β_r_* and *K_r_* can have similar downstream effects [Fig. 2(c)] and specifically interpreting mutations as sources of long-lived (as opposed to transient) changes in these parameters. Then we continue by experimentally verifying the importance of cooperativity in maintaining stable expression of downstream genes in the presence of detrimental mutations to the repressor. Also, by simultaneously following LexA promoter output (mCherry) and target gene output (GFP), we experimentally show a concerted response to increasingly deleterious repressor mutations.

In eukaryotes, understanding the role of negative feedback loops in cell survival and evolution is critical because there are a large number of proteins whose expression is governed by negative feedback loops, including many TFs involved in cancer, such as p53. Defects in negative feedback mechanisms are capable of enhancing proliferative cell signaling that promotes tumorigenesis [47]. In fact, specific disruption of p53’s negative feedback loop results in extreme radiosensitivity [48] and multiple clinical trials are underway that target p53 or its feedback loop [49]. Therefore, the emergence of this mechanism is broadly relevant to human health and to the pursuit of drugs that target negative feedback mechanisms.

## Figures and Tables

**FIG. 1:**
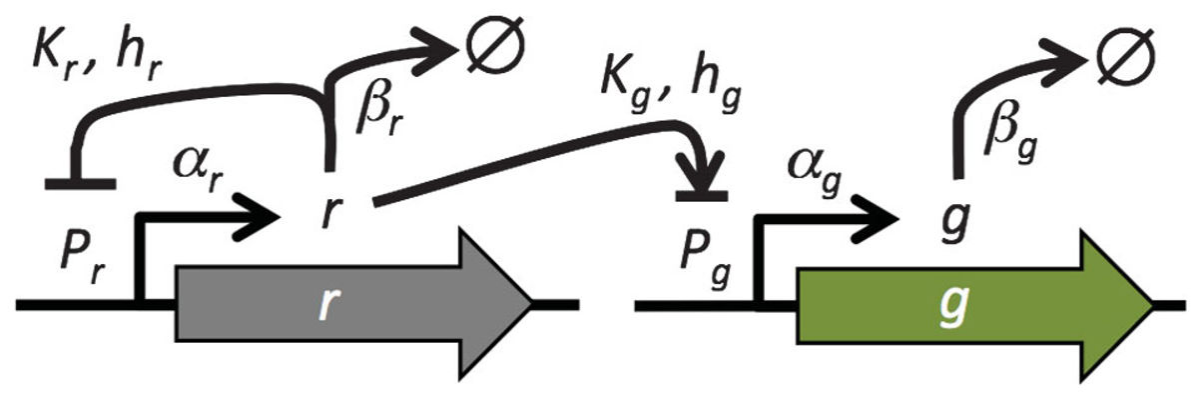
Schematic of the genetic circuit containing the negatively autoregulated transcription factor (gray arrow) and target gene (green arrow). The TF represses its own promoter (*P_r_*) and either represses or activates the promoter of the target gene (*P_g_*). Slashed O sign denotes removal of gene product. Biochemical interpretations of the symbols are given in Table S.1 of the Supplemental Material [18].
